# A Manual of Medical Jurisprudence

**Published:** 1867-02

**Authors:** 


### BIBLIOGRAPHICAL.

*A Manual of Medical Jurisprudence.* By Alfred Swayne Taylor, M. D., F. R. S. Fellow of the Royal College of Physicians, and Professor of Medical Jurisprudence and Chemistry in Guy's Hospital, Sixth American, from the eighth and revised London edition, with notes and references to American Decisions. By Clement B. Penrow, of the Philadelphia Bar.

This work should be in the hands of every physican. Too little attention has been paid to the medico-legal questions so often occurring in courts and elsewhere; and it is painful to witness the ignorance of many medical gentlemen on the subject, who are otherwise well informed. It holds an important place in polite literature, and no man can be considered accomplished without a knowledge of it.

